# Long-Term Effects of Child Early Surgical Ventricular Septal Defect Repair on Maternal Stress

**DOI:** 10.3390/children10121832

**Published:** 2023-11-21

**Authors:** Jennifer Gerlach, Elena S. Decker, Anne-Christine Plank, Stefan Mestermann, Ariawan Purbojo, Robert A. Cesnjevar, Oliver Kratz, Anna Eichler

**Affiliations:** 1 Department of Child and Adolescent Mental Health, University Hospital Erlangen, Friedrich-Alexander-Universität Erlangen-Nürnberg (FAU), 91054 Erlangen, Germany; 2 Department of Pediatric Cardiac Surgery, University Hospital Erlangen, Friedrich-Alexander-Universität, Erlangen-Nürnberg (FAU), 91054 Erlangen, Germany; 3 Department of Pediatric Cardiovascular Surgery, Pediatric Heart Center, University Children’s Hospital, 8032 Zürich, Switzerland

**Keywords:** congenital heart disease, ventricular septal defect, pediatric cardiac surgery, mother, maternal stress, maternal well-being, psychological adjustment, hair cortisol, longitudinal study

## Abstract

The ventricular septal defect (VSD) represents the most common congenital heart defect (CHD). The diagnosis of and cardiac surgery for their child’s VSD are highly stressful experiences for parents; especially mothers, who are at risk of developing long-lasting stress-related symptoms. This study examined long-term alterations in maternal stress including self-reported psychological and biophysiological stress levels in a case-control design. We investigated 24 mothers of children with an isolated, surgically corrected VSD compared to non-affected controls. Maternal self-reports on psychopathology, everyday stress, parenting stress and hair cortisol concentrations (HCC) were measured during children’s primary school age (6–9 years, t1) and early adolescence (10–14 years, t2). In maternal self-reports, psychopathology and stress symptoms in the VSD-group and controls were comparable at t1, whereas at t2, mothers in the VSD-group even showed a decrease in psychopathology. Maternal HCC levels in the VSD-group were significantly lower (hypocortisolism) than HCC levels of controls at t1. This effect was no longer observed at t2 reflecting an approximation of HCC levels in the VSD-group to controls’ levels. This study highlights the potential for improved stress hormone balance and psychological well-being in mothers following their child’s surgical VSD repair. However, the need for parent-centered interventions is discussed, particularly during peri-operative phases and in early child developmental stages.

## 1. Introduction

With an estimated birth prevalence of 0.8–1%, congenital heart defects (CHD) are the most common congenital organ mutations occurring in humans [1]. The term “CHD” includes various types of defects, from mild to severe [2]. Accounting for approximately one third of all CHDs, the ventricular septal defect (VSD) is the most common heart malformation [3]. In this case, there is an opening in the ventricular septum which separates the two heart chambers [4]. Although a universal consensus for the classification of severity does not exist for CHDs, isolated VSD is considered a “simple” form in terms of disease complexity [5]. Thanks to advancements in medical technology, the morbidity and mortality rates of patients surgically treated for VSD have been drastically reduced in the last few decades [1] and the long-term physical outcomes after surgery are described as good to excellent [6]. Although somatic developmental outcomes for children with VSD are expected to be positive, the question of psychosocial adjustment remains for affected children and their families. Only a few studies have focused on children’s psychosocial adjustment after early surgical VSD repair. However, recent findings showed that psychosocial adjustment and high quality of life after an early surgical VSD correction are possible for affected children, if their mothers reported low anxiety levels and a highly proactive and warm parenting style [7,8].

The association of parental well-being and the psychosocial adjustment of children with CHD in general is well documented [9]. Not only CHD and subsequent medical treatment or surgery may have an impact on child development, parental characteristics may also have an additional influence [10]. Parental psychological functioning has been linked to psychopathological symptoms in children who underwent pediatric surgery [11]. In addition, especially in early childhood, children depend on their parents for support in behavioral and emotional regulation as well as in coping with difficult situations such as pediatric cardiac surgery [12], highlighting the important role of parents for children’s development after a cardiac surgery [7,8,13]. Following this line of thought, the psychosocial impact of children’s CHD and cardiac surgery on parents has become an important outcome measure, and research is increasingly including the perspective of parents as well as the stress they experience [10].

The hypothesized underlying mechanism of how parental characteristics can influence child development is that psychosocial stressors—like parental psychopathology or a poor health condition of the child—have a negative impact on the parent–child interaction by affecting parents’ capacity to interact emotionally warm and sensitively with their child. The resulting dysfunctional parent–child interaction, in turn, can have negative consequences for child development [14,15,16]. A pediatric cardiac surgery of the child has already been identified as such a psychosocial stressor: A recent systematic review reported that most studies observed difficulties in the parent–child interaction in families with a child with a CHD [16]. Many studies have linked parental (psychological) stress not only to an altered parent–child interaction (i.e., parenting behaviors like parenting styles or sensitivity) but also to dysfunctional infant brain development, and behavioral, cognitive or socio-emotional impairments in children [13,17]. The parent–child interaction is therefore seen as the stress or risk transmitting variable on child developmental outcomes [18].

Thus, paying attention to parental stress and well-being after a CHD diagnosis and the following process of medical care and pediatric cardiac surgery is crucial (for a summary, see [19]), both for parental well-being and psychosocial adjustment as well as for child development. Following this line of thought, this study focused on maternal stress and well-being after their child’s early surgical VSD correction from children’s primary school age to adolescence, both on the psychological (psychopathology, self-reported stress experience) and biophysiological (hair cortisol) level.

*Psychological stress experience.* The congenital heart disease of their child represents a highly distressing experience for parents [20], involving a wide range of feelings like anxiety, fear, disbelief, helplessness, guilt and grief [21]. In total, 30–80% of parents of a child suffering from CHD report high stress experiences [14]. Research on parental stress in families with CHD-affected children is highly heterogeneous; among other reasons, because of high variability in samples with regard to the severity of CHD. Only a few studies have focused on specific CHDs like (isolated) VSD in homogeneous samples [7]. Moreover, psychological distress of the respective parents has been assessed by a variety of measures as well as being described using different terms, including stress, worry or concern, anxiety, depression and post-traumatic stress disorder (PTSD) [22], so general conclusions on stress levels of parents with children with different types of CHD cannot be made.

However, parental stress can, in turn, impact parental mental health and well-being [9]. From the moment the CHD diagnosis of the child is provided, a phase of increased stress begins for the affected parents, who undergo several peaks in stress experiences during the complete healthcare process from diagnosis to discharge after cardiac surgery [22]. In particular, the pediatric cardiac surgery has been identified as a highly stressful period. Although surgical VSD repair is considered a safe procedure, the general somatic risks of a heart surgery remain [23]. The experience itself can be a highly stressful or even traumatic event for the children involved and their mothers, as pediatric cardiac surgery is a potentially life-threatening situation [24]. Accordingly, in the period after the child’s heart surgery, 80% of the parents reported clinically significant trauma symptoms, with up to 30% meeting the criteria for a PTSD diagnosis [25]. The series of stressful events associated with the child’s CHD might trigger subsequent other psychosocial stressors [26], like marital or financial issues [27], resulting in considerably high levels of distress experienced by parents [28].

Meanwhile, there are findings on the course of the parental stress phase around the time of the child’s cardiac surgery. In the period prior to the child’s cardiac surgery, parental stress levels start to rise [29], followed by high levels of stress and anxiety during the whole peri-operative period [30]. Moreover, affected parents are reported to have an increased risk for mental health issues, particularly in the immediate post-surgery period [25,28]. Studies with longer follow-ups revealed mixed results on parental psychosocial adjustment after their child’s heart surgery. However, to the best of our knowledge, no studies have tracked child development following pediatric cardiac surgery from infancy through adolescence.

Some studies have shown that psychosocial adjustment is possible in the long-term; in the first six months following the child’s cardiac surgery progress, parental stress symptoms were found to decrease [11,29,31]. A similar reduction in parental stress was also found in studies with a longer follow-up period [31,32]. For example, comparable up to even lower stress levels of parents with CHD- and VSD-affected children were detected in comparison to a control group after several years (up to 8 years after cardiac surgery) [33,34,35]. A meta-analysis by Kolaitis and colleagues (2017) indicated that the majority of parents successfully adapted to living with a child with CHD in the long term [14]. According to Doherty and coworkers (2009), better psychological functioning was predicted by coping skills, good understanding of the diagnosis and stronger cohesive family functioning [36].

However, one third of parents continued to experience high levels of stress in the first six months after their child’s heart surgery [29], and approximately 40% of affected parents reported a need for psychosocial care [14]. Mothers have been particularly reported to be at high risk of developing long-term consequences like mental health problems after their child’s heart surgery, and have been found to have higher levels of stress, depression and trauma than fathers [22,25,36,37]. Some studies assumed that mothers might respond to the situation of having a child with CHD by maintaining elevated levels of vigilance [38]. Additionally, it is assumed that mothers experience greater parenting demands and carry a greater caregiving burden as they tend to spend more time nurturing their ill child [25]. This may lead to exhaustion and less resources for positive activities like socialization [37] and may influence mothers’ responsiveness to their child [14].

Furthermore, there is inconsistency regarding the question as to whether the severity of the child’s heart defect influences parental outcomes. Many studies have revealed higher parental stress levels or mental health problems with increasing severity of the diagnosis [22,25,39]. In contrast, some studies have reported no significant predictive value of CHD severity on parental stress measures per se [14,35,36,37,40].

In sum, the existing literature provides ambiguous results on the persistence of stress in parents of a child with CHD. After high stress levels around the time of surgery, there is a tendency to a decreasing impact over the disease course. Nevertheless, the continuously heightened risk of long-term psychological issues for affected parents as well as the consequences of parental stress for the parent–child interaction is pointed out. An important influencing factor is the gender of the parent, with mothers being at greater risk.

What makes the long-term psychological consequences critical is their link to health outcomes. There is extensive literature about the effects of repeated and long-term stress exposure on physical and mental health outcomes and multiple causal pathways have been discussed [41]. One extensively studied pathway is the regulation of the hypothalamic–pituitary–adrenal (HPA) axis and the dynamics of cortisol, its key effector hormone mediating the stress response.

*Biophysiological stress response.* When a threat exceeding a certain severity or time limit is detected by the brain, the compensatory so-called stress reaction is activated [42]. This notably consistent, well-coordinated and generally adaptive response [42] enables individuals to respond appropriately to threats and to navigate their environment [43]. The acute stress response, with a duration of minutes to hours, is therefore referred to as “nature’s fundamental survival mechanism that enhances protection and performance” [44]. All systems of the organism are affected, including endocrine, musculoskeletal, respiratory, cardiovascular, nervous and reproductive systems [45]. Accordingly, the stress response involves a complex orchestration of different, interacting systems [41], such as the sympathetic branch of the autonomic nervous system which mediates immediate adaptations and the HPA axis. Its activation stimulates the production of glucocorticoids, primarily cortisol [46]. Subsequent biophysiological changes including a modulation of the immune system prepare the organism for coping with the acute stressor. Biophysiological stress reactivity to daily stressors can show large interindividual differences and biophysiological responses may differ from subjective stress appraisals [47].

In contrast to time-limited, temporary stress, chronic stress has long been identified as potentially harmful and as a risk factor for the development of various diseases [44]. Although there is plenty of research conducted on both acute and chronic stress, there is less understanding of the turning point in which acute turns into chronic stress and starts affecting the individual’s health in a negative way [43]. In the resting state, stress system activity and cortisol exhibit a distinct diurnal pattern, coupled to the light–dark and sleep–wake cycles. A peak in cortisol in the early morning is followed by a gradual decline over the course of the day [48].

In the acute state of physical or psychological stress, circulating cortisol levels are increased and the adaptive body response is initiated until levels return to a normal state after overcoming the stressor [49]. Moreover, inflammatory mechanisms are stimulated, which might serve protective functions in the short term, but have severe long-term health consequences if sustained [43]. Under the strain of repeated stressful stimuli, the levels of circulating cortisol normally remain elevated over a prolonged period of time [49] and the stress system is chronically hyperactivated [42].

In the long term, alterations in the HPA axis are the result [50]. Extended periods of elevated cortisol, also referred to as hypercortisolemia, are found in persons with depression, anorexia nervosa, generalized anxiety and panic disorder or alcohol withdrawal, among other conditions. After a long-lasting stress exposure, a transition from the initial hypercortisolemic phase to a secondary, compensatory HPA axis downregulation with lowered cortisol production, a hypocortisolemic phase is proposed [42]. This phase is reported to be commonly accompanied by high stress sensitivity, chronic fatigue and chronic pain and has been linked to chronic and traumatic stress exposure [42].

In view of the likely permanent changes in emotional, physiological and behavioral responses, Cohen and coworkers (2019) stated that chronic stress is considered the most toxic form of stress exposure [26]. It surely is debatable, whether VSD diagnosis and correction can be seen as a chronic stressor for the affected parents years after the surgery. What could be of importance in this regard is that prolonged exposure to stress can also derive from anticipatory stress, worry, and post-event rumination. Despite the absence of threat, worry and rumination can elevate baseline levels of physiological arousal, facilitate permanent reactivity and therefore extend the impact of acute stressors. This process is referred to as perseveration. Although not yet quantified, perseverative cognition represents an important mediator of the stress–disease link, as it affects cardiovascular, autonomic, and endocrine nervous system activity. Thus, psychopathological as well as somatic effects are to be expected by “mere thinking about stressful events, from the past or in the possible future” [51]. In consideration of this, it is worth mentioning that mothers of children with CHD have reported maintaining heightened levels of vigilance regarding any health symptoms of their child [38].

*Psychobiological measurement of stress.* Based on the insights of long-lasting research, there is consensus that a single stress-specific biomarker does not exist [52]. However, the extensive literature focuses on the measurement of cortisol, most commonly in body fluids like serum, saliva and urine [53].

For the evaluation of long-term, cumulative cortisol concentrations, cortisol extraction from hair samples is the method of choice [54]. Human scalp hair has a mean growth of 1 cm per month [55]. Despite incomplete understanding of substance incorporation into hair, measurement of hair cortisol concentration (HCC) is increasingly seen as a valid reflection of the average HPA axis activity in retrospective [49], with the HCC measured in the most proximal centimeter corresponding to the cumulative cortisol concentration of the past month. Weak and statistically well-adjustable associations of HCC to demographic, hair-cosmetic and lifestyle factors are reported. Gender is the most robust covariate found, with an overall estimate of 21% higher HCC in men [56].

Overall, chronic stress has been associated with a 22% higher HCC, with even higher concentrations when stress persists at the time of measurement. The tendency of a decrease in cortisol over time to a hypocortisolemic phase, as mentioned before, can also be observed in HCC [56].

Self-report measures of stress only correlate weakly with short-term as well as long-term biophysiological cortisol measures [56,57,58] and are consequently described to represent a different stress concept [59]. Self-report questionnaires provide a more subjective impression and are likely affected by different reference norms and individual biases, as found in many areas of psychometrics [60]. Biomarkers are believed to offer more reliable and precise information [57] and were statistically superior in predicting stress-related outcomes [58]. As both approaches hold valuable information but also methodological challenges [57], an integrative approach with the two complementing each other has often been favored [58].

*Aims of the present study*. Since the early 2000s, the need for further investigation of parents’ struggling to adapt to their child’s illness and cardiac surgery has been highlighted [32]. Even though research identified mothers as an at-risk group for negative long-term consequences (e.g., psychopathology) after their child’s cardiac surgery [22,25,36,37,40], there is still an imbalance in favor of studies addressing the psychosocial development of children with CHD, and findings on their parent’s stress are scarce [20]. The few studies examining the stress-related outcomes of parents of children with CHD often chose self-report measures to capture the family’s long-term psychosocial adjustment. On this behalf, there were calls for the additional use of other measures, such as behavioral or biophysiological ones [22,36]. Moreover, a lack of longitudinal studies with follow-ups through childhood is reported [22]. In addition, the deduction of robust results and recommendations is particularly complicated due to heterogenous samples of most studies and meta-analyses consisting of a variety of CHDs. As different CHDs varying in severity are not necessarily comparable, studies including only a specific type of CHD are needed. Homogenous samples of parents and children who specifically had a surgical correction for VSD have rarely been studied in the long-term. As children are considered somatically healthy after the successful surgery, VSD patients and their families are often lost to follow-ups [7]. Consequently, secondary impairments might be overlooked.

Therefore, this longitudinal study aimed to investigate maternal stress outcomes after their child’s early pediatric cardiac surgery from primary school age into adolescence, especially focusing on a homogenous sample of children with an isolated VSD and their mothers. The stress development over time of affected mothers was compared to a control group of non-affected mothers with typically developing children. Moreover, following a multi-method approach, our study combines both maternal self-reported psychological stress (psychopathology, everyday stress, parenting stress) and biophysiological stress markers (HCC). Hence, we addressed the following two main research aims:

First, we wanted to investigate whether mothers of children with an early corrected VSD show altered self-rated measures of psychological stress (psychopathology, everyday stress, parenting stress) from primary school age (t1) of their child into adolescence (t2), compared to a control group of non-affected mothers. Previous studies were heterogeneous in terms of designs and measures, and revealed ambiguous results; some studies showed comparable or even lower levels of self-reported stress in affected parents’ ratings years after the child’s cardiac surgery compared to controls [33,34,35]. Other findings revealed conspicuities in the perception of control over life [33] and state anxiety [35] in samples with mothers of children with CHDs. However, these samples consisted of families affected by different types of CHDs, lacking specific evidence about families affected by VSD.

Second, we wanted to examine potential differences in the cortisol stress system analyzing hair cortisol levels of mothers whose children underwent early VSD correction in comparison to mothers of non-affected children. To date, there is little research on biophysiological stress measures in this group. HCC as a retrospective measure of long-term stress adds an additional perspective on the maternal cortisol stress system as it represents an accumulated value over the last four weeks and is not subject to daily fluctuations. To our knowledge, and to date, the retrospective stress measure HCC has not been used in a sample of mothers of children with VSD.

## 2. Materials and Methods

### 2.1. Study Design

**Figure 1:**
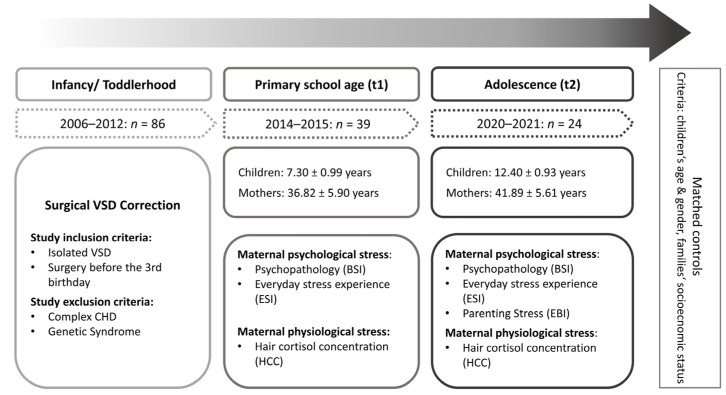
Overview of the study design. Note. BSI, Brief Symptom Inventory [62]; ESI, Everyday Stressors Index [63]; EBI, German version of the Parenting Stress Index [64]; HCC, hair cortisol concentration.

This study is part of a longitudinal research project of the Department of Child and Adolescent Mental Health in cooperation with the Pediatric Cardiac Department at the University Hospital Erlangen, Germany. Children who underwent surgical VSD correction prior to their third birthday and their mothers were assessed at primary school age (t1: 2014–2015) and in adolescence (t2: 2020–2021), and were compared to a non-affected control group. The control group consisted of a general population sample, matched in terms of family socioeconomic status (SES) and child age and gender. Control families were recruited from the Franconian Cognition and Emotion Studies sample (FRANCES) [61]. An overview of the study design is illustrated in Figure 1.

### 2.2. Participants

Between 2006 and 2012, 86 children under the age of three were treated surgically for a VSD in the Department of Pediatric Cardiac Surgery at the University Hospital Erlangen, Germany. In 2015, affected families were contacted regarding participation in a longitudinal study by the Department of Child and Adolescent Mental Health (University Hospital Erlangen, Germany). Due to genetic syndromes (e.g., Down syndrome; *n* = 14), additional congenital malfunctions (e.g., VACTERL association; *n* = 5), complex heart defects (e.g., Tetralogy of Fallot; *n* = 6) and one non-cardiological death, 26 children could not be included in this study. Ultimately, 60 families fulfilled the inclusion criteria. Six families had changed residence and fifteen families were not interested in participating in this study, resulting in a sample of 39 mother–child pairs (65%) at t1. Of the original 39 children and their mothers, 24 attended the second investigation. The other families were either not interested (*n* = 7) or not available anymore (*n* = 8) (38.5% drop-out). The infancy–childhood–adolescence longitudinal sample therefore consisted of 24 mother–child pairs and 24 matched control mother–child pairs [7,8].

At t1, the mothers were between 24 and 46 years old (VSD: 36.82 ± 5.90 years; controls: 39.44 ± 4.64 years), and correspondingly between 30 and 52 years old at t2 (VSD: 41.89 ± 5.61 years; controls: 45.21 ± 4.70 years). Their children were aged 6 to 9 years old at t1 (VSD: 7.31 ± 1.10 years; controls: 7.21 ± 0.69 years), accordingly between 10 and 14 years old at t2 (VSD: 12.40 ± 0.93 years; controls: 13.19 ± 0.24 years). All relevant sociodemographic and health characteristics are reported below (see Table 1), including group difference statistics.

At t2, children in the VSD group were significantly younger than the control group and mothers in the VSD group were significantly younger than mothers in the control group. The distribution of male and female children did not differ between the groups. Moreover, there were no significant differences in migration background, families’ SES, number of children under 18 in the household, working hours per week, and the incidence of maternal psychiatric diagnoses between the control and VSD group.

### 2.3. Procedure

As already mentioned, the study design contained two assessment points, one at child primary school age (t1) and one in adolescence (t2). At both times of measurement, beyond others, mothers were interviewed for socioeconomic data, own health and medication status during a 2-h interview. Information regarding own psychopathology and everyday stress was gathered using standardized questionnaires. In addition, hair strains were cut.

Due to the COVID-19 pandemic, families were offered different participation modes at t2: Participation face-to-face, home visits and contact-free participation via mail. While *n* = 16 mothers and children re-participated in person at the Department of Child and Adolescent Mental Health, *n* = 4 preferred to be visited at home and another *n* = 4 participated via mail. The current study protocol was approved by the Local Ethics Committee of the Faculty of the University of Erlangen-Nürnberg (t1: 4596, 2 April 2014; t2: 353_18B, 12 April 2019) and conducted in accordance with the Declaration of Helsinki. Mothers gave written informed consent and the assent of the children was obtained.

### 2.4. Measures

*Family Sociodemographics.* Information on family demographics and life circumstances was collected during an interview. A socioeconomic status (SES) sum index with a theoretical range from 3 to 16 was calculated, higher values indicating a higher family SES. The index combines the family income per month (six-staged (1–6): EUR < 1000, 1000–2000, 2000–3000, 3000–4000, 4000–5000 and >5000 per month) as well as the education level (four-staged (1–4): <9, 9, 10, or 13 years of education) and the migration background of both parents (two-staged: yes (0) vs. no (1)) [7]. In the present sample, SES ranged from 6 to 16 (11.35 ± 2.51).

*Maternal Psychopathology.* Maternal psychopathology was assessed using the German version of the Brief Symptom Inventory (BSI) [62,65]. In this 53-item self-report measure, clinically relevant psychological symptoms regarding the last seven days are rated on a 5-point Likert scale (0 = “not at all”, 1 = “a little bit”, 2 = “quite a bit”, 3 = “highly”, 4 = “very much”). Participants are provided with lists of symptoms and were asked how much they were distressed by these symptoms in the last seven days. Examples for symptoms are “faintness or dizziness”, “poor appetite”, “feeling inferior to others”, “feeling fearful” or “feeling hopeless about the future”. Nine symptom dimensions are covered: Somatization, obsession–compulsion, interpersonal sensitivity, depression, anxiety, hostility, phobic anxiety, paranoid ideation and psychoticism. For this analysis, the Global Severity Index (GSI) was calculated by summing all item scores, dividing them by the total number of items to which the participant responded and transforming the raw values into T-scores according to norm tables (T-scores: 50 ± 10). The higher the resulting T-score, the higher the respondent’s stress level due to psychopathological symptoms. Two thirds of the norm sample’s T-scores lay between 40 and 60. The author suggests considering scores from 63 clinical cases. While the internal consistency of the nine scales varied between α = 0.39 and α = 0.89. in different reliability tests, consistently high values were found for the GSI (α = 0.92–0.96). Retest reliability (*r* = 0.68–0.93) was good and sufficient evidence speaks for the construct validity of the scale structure [62].

*Maternal Everyday Stress.* The German adaptation of Hall’s Everyday Stressors Index (ESI) [63,66] was included in this study to collect information about maternal everyday problems and stress. The 18 four-point Likert scale items, drawn from the Hassles Scale [67], concern financial and interpersonal problems, job problems, role strain and parental worries. The items consist of problem statements such as “Owing money to others or incurring debts”, “Unsafe living environment” or “Difficulties with the father of your child/children”. The rating scale ranges from 1 for “does not bother me at all” to 2 for “bothers me a little” and 3 for “bothers me a little more” to 4 for “bothers me a lot”. As individual ratings are summed up to an overall value, the total score ranges between 18 and 72 with higher scores indicating higher subjective burden due to daily hassles. Internal consistency is reported to be high (α = 0.86).

*Parenting stress.* The measurement of parenting stress was conducted using the German version of the Parenting Stress Inventory (PSI; German version: Eltern-Belastungs-Inventar (EBI)) [64], a questionnaire for parents with the aim of identifying different sources and domains of stress in parenting. A total of 48 items in 12 subscales assess the child’s behavior and characteristics as well as impairments in the parental function. The subscales of the child domain are Distractibility or Hyperactivity of the Child, Acceptability, Demand, Adaptability and Mood, whereas the parental domain contains the subscales Attachment, Social Isolation, Doubt in Parental Competence, Depression, Health, Personal Restriction and Partner Relationship. Exemplary items are “Being a mother/father is more difficult than I thought” or “My child is often unfocused and easily distracted”. Respondents rate their approval with the statements on a 5-point Likert scale (1 = strongly disagree, 2 = somewhat disagree, 3 = neither agree nor disagree, 4 = somewhat agree, 5 = strongly agree). A total score can be conducted by adding up all raw values and using an age-independent norm table to convert raw values into standardized T-scores (50 ± 10). In addition, scores of the child and parent domain can be determined separately. Higher scores reflect higher parenting stress in the respective domains. Validity, internal consistency (α = 0.95) and retest reliability (r = 0.87; after one year) were verified.

*Hair Cortisol Concentration (HCC).* To determine the cumulative cortisol level over the period of the last month before sampling, a strand of hair, approximately 0.5 cm wide, was cut posterior as close to the hairline as possible. The first proximal centimeter was used for analysis, as hair grows approximately 1 cm per month. The samples were stored at +4 °C until further analysis. Mothers were interviewed about chemical hair treatments, medication and oral contraceptive intake.

Cortisol was extracted from samples collected at t1 as described by Grimm and colleagues (2021) [68]. Briefly, samples were incubated with 2 mL methanol for 24 h, the methanol supernatant was transferred into a fresh tube and evaporated at 60 °C overnight to obtain a dry, methanol-free pellet. This pellet was then dissolved in phosphate-buffered saline (PBS) and stored at 4 °C until analysis. To the samples collected at t2, an improved extraction method was applied [69]. After washing, drying, weighing and grinding, the extraction procedure was extended to a 4-step process in order to improve the cortisol extraction. During the washing procedure, samples were incubated twice with 2.5 mL isopropanol for 3 min at room temperature. The hair samples were then transferred into cryovials containing steel grinding balls and subsequently air dried at room temperature for at least two days. The hair was minced in a ball mill (Retsch GmbH, Haan, Germany) for five minutes, and the weight of each sample was determined by subtracting the tare weight from the gross weight. The 4-step cortisol extraction method comprised an incubation step with 1 mL of methanol for 24 h, followed by centrifugation (10,000× *g*, 2 min) and separation of the supernatant, a second incubation step with 1 mL of acetone for 5 min (again followed by centrifugation and separation of the supernatant), and one repetition of both steps. Finally, the pooled methanol acetone supernatant of each sample was evaporated at 50 °C. The resulting pellet was stored at 20 °C until analysis and dissolved in 250 µL PBS immediately before analysis.

HCC protein concentrations were determined via Bradford Protein Assay (Roti-Quant Protein quantitation assay according to Bradford; Carl Roth GmbH, Karlsruhe, Germany). Cortisol concentrations were measured using salivary cortisol ELISA kits (t1: KA1885; Abnova, Taipei, Taiwan; t2: RE52611; IBL International, Hamburg, Germany) according to the manufacturers’ instructions. Each sample was assayed in duplicate (Benchmark Plus microplate spectrophotometer; Bio-Rad Laboratories, Hercules, CA, USA) and the mean values and coefficients of variation (CV) were computed for each duplicate.

Each HCC was normalized to sample protein concentration (t1)/sample weight (t2) and ln-transformed (since standardized HCC were not normally distributed) as follows:

$$t1:\;\mathrm{Cortisol}\text{-}\mathrm{to}\text{-}\mathrm{protein}\;\mathrm{ratio}\;(\mathrm{pg}/\mathrm{mg})=\ln\left(\frac{HCC\left(\frac{ng}{mL}\right)}{HPC\left(\frac{µg}{mL}\right)}\times 10^{6}\right)$$

$$t2:\;\mathrm{Cortisol}\text{-}\mathrm{to}\text{-}\mathrm{weight}\;\mathrm{ratio}\;(\mathrm{pg}/\mathrm{mg})=\ln\left(\frac{HCC\left(\frac{ng}{mL}\right)\times 0.25mL}{hairsampleweight\left(mg\right)}\times 1000\right)$$

Valid hair cortisol data were available for 23 of these mothers (VSD: *n* = 11; controls: *n* = 12), as cases had to be excluded due to missing cases (*n* = 3) or exclusion criteria (*n* = 22). Reasons for exclusion were: Coefficients of variation (CV) exceeding 0.20 (t1: *n* = 4, t2: *n* = 4); outlier values identified via z-standardization [70] (*SD* ± 3.29; t1: *n* = 3, t2: *n* = 3); hair treatment causing visible discoloration of the sample during the cortisol extraction process (t1: *n* = 0, t2: *n* = 1); and medication intake including corticosteroids, beta-blockers [24] and psychopharmaceuticals (t1: *n* = 5, t2: *n* = 7) [71]. Samples meeting more than one exclusion criterion were mentioned multiple times. Hair treatment per se was not defined an exclusion criterion but was tested as a potential confounder together with age and oral contraceptive intake.

### 2.5. Statistical Analyses

Analyses were performed with the statistical software IBM SPSS Statistics (version 29.0.1.0; Armonk, NY, USA: IBM Corp., 2018). Descriptive statistics were conducted and normal distribution was analyzed via quantile–quantile plots and Shapiro–Wilk tests. Group differences between VSD and control group in sociodemographic and mental health characteristics were tested with Chi-squared tests and independent *t*-tests. Mann–Whitney-U Tests were additionally conducted for variables deviating from normal distribution, but as results did not differ from parametrical tests, results of the parametric tests were reported. Intercorrelations of the psychological stress measures, BSI, ESI and EBI, as well as the biophysiological measure of hair cortisol were investigated with Pearson’s correlation (*r*).

For examining group differences in stress markers between VSD (*n* = 24) and the control group (*n* = 24) over the course of the two measurement times, repeated measure analyses of variance (rm ANOVAs) were conducted for two of the psychological stress measurements, BSI and ESI, as well as for biophysiological stress HCC data. When rm ANOVA indicated a main group (VSD vs. controls) or interaction (group × time) effect, Bonferroni-corrected post-hoc tests (F-Tests) were conducted. As EBI was only measured at t2, a *t*-test for independent samples was conducted.

Before rm ANOVA testing, HCC data were prepared for statistical analysis. Cases with applying exclusion criteria were excluded (see the paragraph on HCC in the methods section). Due to differences in HCC sample methods between t1 and t2, values from t1 and t2 were separately z-standardized to ensure comparability. Moreover, the potential confounders of age, hair treatment and oral contraceptive intake were tested with Pearson’s correlation for both times of measurement. The variables of age, hair treatment and oral contraceptive intake were not significantly associated with HCC and were therefore not included in the hypothesis testing. In the case of Pearson’s correlation and rm ANOVAs, deviations from the normal distribution were considered negligible in view of the robustness of the procedures against violation of the normality assumption [72].

For all analyses, the significance level was defined as *p* < 0.05 (two-tailed). Considering the small sample size, results with a level of significance of *p* ≤ 0.10 were interpreted as trends. Pearson’s correlations were interpreted as small (│*r*│ = 0.10), medium (│*r*│ = 0.30) and strong (│*r*│ = 0.50) associations. Phi-coefficients, as effect size measures in χ^2^-tests, were classed as small (│φ│ = 0.10), medium (│φ│ = 0.30) and large effects (│φ│ = 0.50). Partial eta-squared (*η*^2^*_p_*), as an effect size measure in rm ANOVAs, and Cohen’s d for post-hoc *t*-tests and effect size measure in *t*-tests, were interpreted as 0.01 ≤ *η*^2^*_p_* ≤ 0.05 and 0.20 ≤ *d* ≤ 0.49 small effect, 0.06 ≤ *η*^2^*_p_* ≤ 0.13 and 0.50 ≤ *d* ≤ 0.79 medium effect and *η*^2^*_p_* > 0.13 and *d* > 0.79 large effect [73].

## 3. Results

### 3.1. Stress Measures Intercorrelations and t1–t2 Stability

The majority of psychological stress measures were significantly intercorrelated (see Table 2). Investigating maternal psychopathology and every day stress t1–t2 stability resulted in significant positive correlations with medium effect sizes. The biophysiological measure HCC was positively related over time, but the correlation did not reach significance and the association was small.

### 3.2. Long-Term Effects of Child Early Surgical VSD Repair on Maternal Stress

*Maternal self-reported psychological stress.* First, we investigated whether mothers showed altered self-rated measures of stress (maternal psychopathology, everyday stress experience, and parenting stress) years after the surgery, compared to a control group.

**Figure 2:**
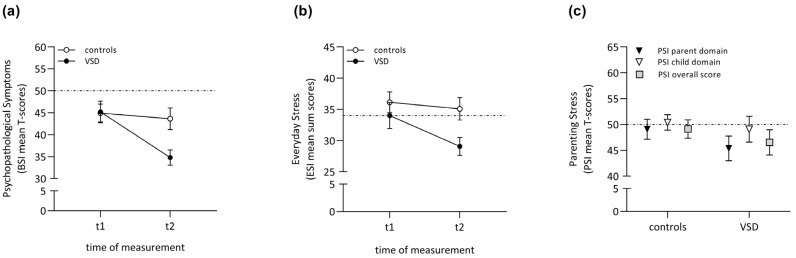
Maternal self-reported psychological stress both in VSD and control group. Note. (**a**) psychopathological symptoms, (**b**) everyday stress, (**c**) parenting stress. The horizontal broken line indicates the norm sample means for reference.

*Psychopathology*. Concerning the psychopathology scores, the rm ANOVA revealed a large-size main effect of time (*F*(1, 45) = 14.11, *p* < 0.00, *η*^2^*_p_* = 0.24), demonstrating a significant difference in maternal psychopathological symptoms between child primary school age (t1) and adolescence (t2). Moreover, a large-size interaction effect between time and group was found (*F*(1, 45) = 8.62, *p* < 0.00, *η*^2^*_p_* = 0.16). Post-hoc tests specified that there was a significant decrease (*p* < 0.001) in maternal psychopathological symptoms over time only in the VSD group (*MDiff* = 10.52, 95%-CI [6.00, 15.05]). Accordingly, the groups differed significantly in psychopathology at t2 but not at t1 (t1: *F*(1, 45) = 0.01, *p* = 0.94, *η*^2^*_p_* = 0.00; t2: *F*(1, 45) = 8.56, *p* < 0.00, *η*^2^*_p_* = 0.16), with mothers in the VSD group having lower psychopathology scores than controls when their children were in adolescence (see Figure 2a). No significant main group effect was detected.

*Everyday Stress*. For everyday stress scores, the rm ANOVA showed a medium-size main effect of time by trend (*F*(1, 45) = 3.93, *p* = 0.05, *η*^2^*_p_* = 0.08), indicating a decrease in maternal everyday stress from child primary school age (t1) to adolescence (t2) over the two groups. Group membership did not significantly interact with time. A medium-size main group effect existed on a level of marginal significance (*F*(1, 45) = 3.42, *p* = 0.07, *η*^2^*_p_* = 0.07), suggesting an overall lower level of everyday stress in the VSD group (see Figure 2b).

*Parenting Stress*. As the measurement of parenting stress was only conducted at the second data collection during adolescence (t2), group differences were examined using independent *t*-tests. No significant group differences were found for parenting stress in adolescence (t2) regarding the overall score, parent domain or child domain (see Figure 2c).

**Figure 3:**
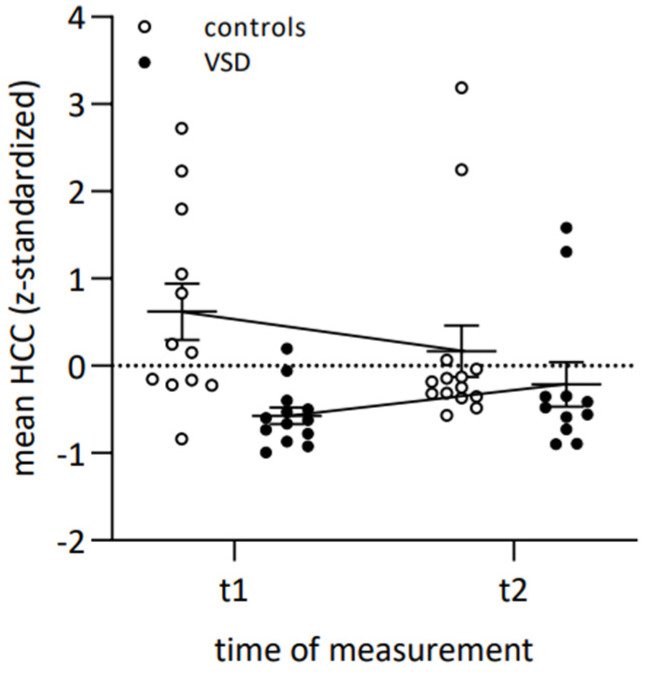
Mean hair cortisol concentration (z-standardized) shown separately for VSD and control group. Note. The horizontal dashed line represents the mean HCC (z-standardized).

*Maternal biophysiological stress.* Second, we were interested whether mothers of children with early surgically corrected VSD would show an altered activity of the cortisol stress system, operationalized by 1-month HCC, compared to the control group. The rm ANOVA showed no significant main effect for time. Results revealed a medium-size interaction effect of time and group that could not reach significance (*F*(1, 21) = 2.72, *p* = 0.11, *η^2^_p_* = 0.12). In view of the small sample size, the medium effect size in the interaction gave reason to consult post-hoc tests. Post-hoc tests specified that the VSD group showed significantly (*p* < 0.001) lower HCC values only at t1 (*MDiff* = 1.31, 95%-CI [0.60, 2.02]). At t2, HCC values did not differ anymore between the two groups. Descriptively, the values approximated each other without significant change from t1 to t2 (see Figure 3). In addition, there was a significant large-size main effect of group (*F*(1, 21) = 9.29, *p* < 0.01, *η^2^_p_* = 0.31) with lower HCC values in the VSD group than in controls.

## 4. Discussion

In this study, we investigated the long-term effects of children’s early surgical ventricular septum closure on maternal stress. Therefore, we examined subjective psychological stress measures (psychopathology, everyday stress, and parenting stress) in combination with biophysiological stress levels (hair cortisol) to provide a comprehensive insight into the maternal stress system.

*Maternal self-reported psychological stress.* Our findings revealed that in both groups, all maternal psychological stress measures varied in a normal, subclinical range.

Regarding maternal psychopathological symptoms, only mothers of children who underwent surgical VSD correction showed a significant reduction in psychopathology from child primary school age to adolescence, whereas the psychopathological symptoms of the control mothers remained stable over time (on a subclinical level). In addition, mothers in the VSD group reported significantly fewer psychopathological symptoms than the control group when their child was in adolescence. Moreover, mothers of children with an early surgically corrected VSD were found to have a lower level of everyday stress at child primary school age and in adolescence as mothers in the control group, by trend.

Generally, the result of maternal subjective, psychological stress levels varying in a normal range fits the former findings of an overall favorable adjustment of children affected by CHDs and their mothers [7,31,32,33]. Due to the children’s healthy and unimpaired life in the years after VSD closure, there might be no reason for long-term subjective stress alteration, despite the stressful or potentially traumatic experiences like VSD diagnosis, medical treatment and pediatric cardiac surgery in the past. According to the maternal self-reports, mothers seemed to be able to adapt to normal (self-reported) stress levels when their children were no longer at direct health risk, which might be especially the case with mild CHDs like VSD. Our findings are consistent with studies reporting on similar outcomes of lower long-term distress in mothers of children with CHDs compared to non-affected controls [34,39]. There might be at least two explanations:

The first explanation is that mothers’ reference norms for stressful events might have been adjusted in the sense that enduring the frightening and stressful period during VSD diagnosis and surgery makes subsequent stressors seem less distressing. The experience of overcoming this impactful period might have made mothers more resilient, potentially changing their values, attitudes and internal representation of stress and problems [34]. In a previous study, part of our research project, the absence of differences in psychopathology and everyday stress experience self-ratings was also discussed to be connected to these adjusted reference norms [24].

A second explanation to consider is parental “stress-related growth” [74] after the journey of VSD diagnosis followed by medical treatment and pediatric cardiac surgery. Stress-related growth is defined as the experience of positive change resulting from highly challenging life circumstances including an increase in personal strength, appreciation for life, meaningful relationships or spirituality [75]. Recent meta-analytic findings suggest that stress-related growth commonly occurs in parents of children with serious pediatric illnesses [76] and was also found in parents having a child undergoing surgery for CHD [17]. To date and to our knowledge, no specific studies focusing on stress-related growth in families confronted with a VSD diagnosis of their child are known. As our study was not designed to measure stress-related growth, it can only be assumed that stress-related growth could be an explanation for lower maternal psychopathology and subjective everyday stress scores; this issue should be addressed further in future studies.

In addition to the longitudinal effects on maternal psychopathology and everyday stress experience, we also assessed mothers’ self-reported parenting stress when their child was in early adolescence resulting in no differences between the VSD and the control group. In contrast, for children with complex CHDs, their parents have shown to experience higher parenting stress than parents of non-affected children [77]. As VSD is considered a more mild form of CHDs, the lack of additional parenting stress might indicate a normative development of the children in adolescence, as we found in our sample published elsewhere [7,8]. The normative development of children who underwent pediatric cardiac surgery due to a VSD could have resulted in comparable parenting stress levels of affected mothers and mothers of typically developing children. Moreover, the time that has passed since the child’s surgical VSD repair may also play a role. Again, watching the child growing up healthy many years after the stressful peri-operative period of VSD correction might have led to adjusted reference norms or stress-related growth in affected mothers resulting in normal parenting stress levels.

In sum, adjusted personal reference norms and stress-related growth effects could be an explanation for lower subjective, psychological stress measures in our sample of mothers in the VSD group. Our findings of overall normal to lower psychological stress measures during child primary school age into adolescence can be seen as an indication for coping success in mothers affected by early child VSD closure over time. Although our findings highlight the opportunity for mothers to show long-term psychosocial adjustment years after their child’s surgical VSD repair, the pre-, peri-, and postoperative periods are still reported to be highly stressful and potentially traumatic [25,30]. Taken together with the results of our study, this suggests that long-term psychosocial adjustment is possible for affected parents, and that interventions, especially throughout the surgical process, may be crucial, but may no longer be necessary in the child’s primary school years through early adolescence.

In clinical practice, it is relevant that the period of increased stress for parents of children with CHDs begins as soon as the diagnosis is made [22]. This means that parents need support from the moment of diagnosis, both in terms of information about the necessary medical care and psychological support to cope with this stressful life event. Psychological support can be provided, for example, by psychologically trained staff in the clinic, but also by parent support groups or parent initiatives [78]. As the peri-operative period of child pediatric cardiac surgery appears to be particularly stressful for parents [30], it would be crucial to provide psychotherapeutic interventions throughout the surgical process. Given the potential impact of subjective well-being on individual health and longevity [79] and the effects of maternal mental health on child development [18], this is of special importance.

Future longitudinal research should ideally add the measurement of stress and psychological adjustment or coping during and shortly after the child’s cardiac surgery, both for gathering more insight in the process of psychosocial adjustment as well as for identifying potential risk and protective factors predicting parental coping. This information can be used for the development of tailored interventions and psychological support for affected parents.

*Maternal biophysiological stress.* To gain a holistic perspective of maternal stress levels, we added the biomarker of hair cortisol in terms of biophysiological stress levels to complement the psychological measures. At child primary school age, we detected lower hair cortisol levels in mothers of children who underwent early surgical VSD repair than in controls. When children were in early adolescence, this difference was no longer apparent; values approximated without significant changes over time.

As stated previously, child chronic illness and surgery are adverse life events and stressful experiences for affected parents. Research on the association of adversity and cortisol resulted in varying results, and the question, if adversity is related to an increase or decrease in HCC, cannot easily be answered [80]. It is currently proposed that after stressful events, extended periods of elevated cortisol or hypercortisolism, transition to a secondary HPA axis downregulation with lowered cortisol production, or hypocortisolism, in the long term [42]. Accordingly, the recent onset of stressful or potentially traumatizing events are considered to be associated with higher hair cortisol levels, while exposure to stressful events that happened a longer time ago have been linked to lower hair cortisol concentrations [81]. Both altered cortisol levels, decreased as well as increased HCC, have been linked to negative psychological and pathological outcomes. In contrast, stable HCC is expected in healthy individuals [82].

Our finding of lower HCC values of mothers in the VSD group at child primary school age compared to the control group and later comparable HCC values in early adolescence fit the current assumption of a long-term hypocortisolemic phase. Our findings show how the cortisol dynamics might develop in the more distant future. If the VSD diagnosis of the child was a stressful experience for affected mothers, lower cortisol at child primary school age could be the last remnant of the hypocortisolemic phase following adverse life events, as found in the literature [42]—while self-reported stress measures were already adjusted. Hypocortisolism has shown to be a risk factor for the development of diseases such as metabolic syndrome, chronic fatigue and pain syndromes, autoimmune diseases and mood disorders [83]. However, beneficial effects of hypocortisolism are also suggested in the literature. Fries and colleagues (2005) argued that it might have a protective function for the organism by reducing harmful allostatic load through reduced HPA axis reactivity [84]. Consequently, the brain might be protected from the effects of chronically elevated cortisol.

Moreover, our results revealed an approximation of HCC levels between the VSD and control group in child adolescence. We interpret this finding as a completed normalization of cortisol levels and the end of the hypocortisolemic phase in affected mothers from child primary school age to early adolescence. In addition, this finding indicates that the normalization of cortisol levels had begun before the first measurement. The literature suggests that the transition from hypercortisolism to a hypocortisolemic pattern might take place after individuals have left the stressful or potentially traumatizing situation [85]. Accordingly, as in the present study, the timespan between the stressful event of the child’s pediatric cardiac VSD surgery and measurements was over several years; therefore, the transition from hyper- to hypocortisolemic phase could have already taken place.

*Associations of self-reported psychological and biophysiological stress measures.* Subjective self-report measures of mothers’ psychological stress were weakly or non-correlated to HCC. Non-existent to weak relationships between self-reports and biophysiological stress measures are well-documented in the literature and might be caused by assessing different constructs related to stress [56,57,58]. The divergence between subjective and biophysiological measures is recognized in many areas of psychometrics [60]. One explanation for this divergence is that subjective stress appraisals are susceptible to biases. Alternatively, altered HCC-levels at child primary school age in combination with normal levels of psychological stress at both times of measurement might again indicate adjusted reference norms or stress-related growth in affected mothers. After the stressful peri-operative period of the child’s surgical VSD closure, maternal internal representations of everyday stress and other problems might have adjusted so that mothers’ self-reports of psychological stress were comparable to non-affected controls; comparable findings were already published for t1 [24]. The biophysiological stress system seems to take a longer recovery time compared to self-reported psychological stress as we found hypocortisolism in maternal HCC scores at child primary school age, which disappeared until early adolescence. Stonawski and colleagues (2018) concluded that altered HPA axis activity could indicate an invisible, but still existing, long-term risk for mothers’ health years after the child’s pediatric cardiac surgery [24]. As maternal (mental) health and well-being is crucial for child development, both in CHD-affected as well as in other risk samples [7,8,17,18], understanding the process of maternal psychological and biophysiological adjustment is of special importance. Since—to our knowledge—no other study has examined the longitudinal biophysiological adjustment of the HPA axis in combination with psychosocial adjustment in mothers with children who underwent pediatric cardiac surgery from primary school age to early adolescence, these hypotheses highlight the need for more research in this area.

*Strengths and limitations.* Since most studies have focused on samples affected by heterogenous CHDs and thus predictions for specific heart defects are difficult to make, the focus on the specific CHD of VSD can be considered a strength of this study. Simultaneously, including only families affected by VSD limited the number of potential participants. Recruiting all participants from the same hospital represents both a strength and a limitation at the same time. It can be assumed that all families received comparable care and support at the hospital and that can be interpreted as standardization; however, it can also be argued that the sample might be selective. Therefore, the generalizability to other populations must be verified.

Further strengths of this study include the matched control group and the longitudinal design. The matched control group improves the control of confounding parameters and supports the identification of statistically significant effects even with smaller sample sizes, and thus increases the statistical power. The longitudinal design is crucial for observing developmental psychological processes and the stability of effects.

Moreover, both self-reported psychological and biophysiological stress measures were included. Hair cortisol was studied for the first time in this specific context, involving a complex laboratory procedure. Hair sampling was highly standardized and by sampling the 1 cm of hair closest to the scalp, it is presumed that environmental influences were minimized. The methods for cortisol extraction and determination differed between t1 and t2, as the extraction process was extended to a four-step procedure at t2 and HCC was normalized to sample weight instead of sample protein concentration. This change was made with the aim to use the interim gain in knowledge and obtain the highest data quality possible. Hair sample weight has recently been considered a better predictor and thus a more suitable reference value for HCC [69]. In the study by Frisch and colleagues (2022), cortisol-to-weight and cortisol-to-protein ratios exhibited a high correlation, indicating that, albeit not ideal, the ratios can be compared [69]. The limited comparability was taken into account by carrying out ln- and z-transformations of the HCC ratios. Since many variables come into question as confounders of HCC, not all potential confounders were controlled, in favor of the test power. The relevant confounders of sex, age, hair treatment and oral contraceptive intake were tested and individuals taking medication with a potential influence on HCC were excluded from HCC analyses.

Another issue to discuss is the focus on only maternal stress-related measures in this study. From a systemic perspective, both parents are highly relevant for child developmental outcomes, and in mental health research, the role of fathers for children’s well-being after a pediatric cardiac surgery is often overlooked [15]. Thus, in future studies, it would be important to include the perspective of the father or the other secondary caregiver.

As mentioned above, several aspects of the research design contributed to the small sample size, including the specific sample, drop-out and exclusion criteria. Consequently, the results and statistical power were limited by the small sample size of *N* = 48, *N_HCC_* = 26, respectively. This was accepted in favor of the benefits and the intention of the project. With this in mind, the results should be interpreted with caution and larger sample sizes should be aimed for in the future.

## 5. Conclusions

To date, and to the best of our knowledge, little was known about long-term effects on maternal stress after children’s early pediatric cardiac surgery in a homogenous sample of children with isolated VSD. In sum, our findings of lower or comparable levels of self-reported psychological stress in several domains as well as the normalization of HCC levels of affected mothers in their child’s early adolescence add a new perspective on the potential for parental psychosocial adjustment and an improved stress hormone balance after the stressful life event in terms of early child cardiac surgery. Thus, our study describes a favorable adjustment and long-term prognosis for mothers of children who underwent early VSD repair. Combined with favorable health prognoses for the affected children [7,8], this message can provide relief and hope for families upon receiving the diagnoses. Nevertheless, the indication of favorable long-term development and psychosocial adjustment of mothers in our sample does not imply that affected families do not need special care in the pre-, peri- and post-surgical period as the process of diagnosis, surgery and post-surgical care still represents a highly onerous and challenging experience [25,30]. To conclude, there is a high need for psychological support and interventions to alleviate the distress of parents especially during the highly stressful period of the child’s cardiac surgery or at early developmental stages. Years after the child’s surgical VSD closure, our findings clearly showed that psychosocial adjustment is possible for affected mothers with somewhat different developmental trajectories of psychological and biophysiological stress. Meanwhile, the self-reported psychological stress of affected mothers already varied in a normal range at child primary school age and even showed a decrease in early adolescence, the biophysiological stress system seems to need a longer recovery time with altered HCC-levels (hypocortisolism) at t1 and normal HCC-levels at t2.

As mentioned in the beginning, the parental experience of stress related to the CHD has both consequences on their own well-being and mental health, but also on children’s developmental outcomes [16], emphasizing the moderating role of parental well-being and mental health on the psychosocial prognosis of the CHD-affected children [7,8,13]. As parents of children with CHD have been shown to benefit from coping skills, a good understanding of the diagnosis and strong cohesive family functioning during the process of medical treatment [36], the importance of early intervention and psychological support of affected parents is stressed [86]; both for the psychosocial adjustment of parents themselves and for the healthy development of their children.

## Figures and Tables

**Table 1:** Sociodemographic and health characteristics of VSD and control group with group difference statistics.

		VSD (*n* = 24)	Controls (*n* = 24)	VSD vs. Controls		
Sociodemographic Characteristics		*M (SD)/N (f(%))*	*M (SD)/N (f(%))*	*t/* *χ^2^*	*p*	*d/* *φ*
Mother age at t1		36.82 (5.90)	39.44 (4.64)	1.68	0.10	0.50
Mother age at t2		41.89 (5.61)	45.21 (4.70)	2.15	0.04 *	0.64
Child age at t1		7.31 (1.10)	7.21 (0.69)	0.41	0.69	0.12
Child age at t2		12.40 (0.93)	13.19 (0.24)	4.04	<0.00 *	1.17
Child gender	Female	13 (54.2%)	14 (58.3%)	0.09	0.77	0.04
	Male	11 (45.8%)	10 (41.7%)			
Migration background		2 (8.3%)	2 (8.3%)	0.00	1.00	0.00
SES		11.08 (2.56)	11.63 (2.50)	0.74	0.46	0.21
Number children <18 y in the household	1	5 (20.8%)	5 (20.8%)	0.51	0.62	0.15
	2	12 (50.0%)	14 (58.3%)			
	3	3 (12.5%)	5 (20.8%)			
	- ^a^	4 (16.7%)	-			
Mother working hours/week		26.09 (11.58)	26.23 (9.06)	0.04	0.97	0.01
Mother mental health (self-report)						
Psychiatric diagnosis		4 (16.7%)	3 (12.5%)	0.17	0.68	0.06
Psychopathology t1		45.2 (11.8)	44.9 (10.0)	0.08	0.94	0.02
Psychopathology t2		34.8 (8.5)	43.6 (12.0)	2.93	<0.00 *	0.85
Everyday Stress t1		34.0 (10.0)	36.1 (8.2)	0.80	0.43	0.23
Everyday Stress t2		29.1 (7.0)	35.1 (8.8)	2.61	0.01 *	0.75
Parenting Stress	Parent Child Overall	45.4 (11.7) 49.1 (12.2) 46.5 (12.0)	49.1 (9.5) 50.4 (7.3) 49.1 (8.6)	0.86 1.21 0.45	0.40 0.23 0.66	0.25 0.35 0.13

Note. Continuous variables are listed as means (*M*) with standard deviations (*SD*), group differences were tested with independent *t*-tests with Cohen’s d indicating effect size. T-values are displayed as absolute values. Categorical variables are listed as *n* (%), and group differences were tested with χ^2^-test (df = 1) with φ-coefficient used as a measure of effect size. SES: socioeconomic family status, additive combination of parental migration background, education level and the family income (theoretical range 3–16); BSI, Brief Symptom Inventory [62], global severity index is indicated (T-score, 50 ± 10); ESI, Everyday Stressors Index [63], sum-score is indicated (theoretical range: 18–72); EBI, German version of the Parenting Stress Index [64], parent domain, child domain and overall (T-score, 50 ± 10). ^a^ missing data. * *p* < 0.05.

**Table 2:** Stability and intercorrelations of the psychological and biophysiological stress measures.

	1.	2.	3.	4.	5.	6.
*Maternal self-reported psychological stress*						
1. Psychopathology t1						
2. Psychopathology t2	0.45 **					
3. Everyday stress t1	0.48 ***	<0.00				
4. Everyday stress t2	0.23	0.50 ***	0.40 **			
5. Parenting stress t2	0.39 **	0.54 ***	0.30 *	0.36 *		
*Maternal biophysiological stress*						
6. HCC t1	−0.05	0.32	<0.00	0.04	−0.01	
7. HCC t2	0.22	0.14	0.31	0.04	−0.01	0.17

Note. Psychopathology: BSI, Brief Symptom Inventory [62]; Everyday stress: ESI, Everyday Stressors Index [63]; Parenting stress: EBI, German version of the Parenting Stress Index [64]; HCC, hair cortisol concentration. * *p* < 0.05. ** *p* < 0.01. *** *p* < 0.001.

## Data Availability

The data presented in this study are available on request from the corresponding author. The data are not publicly available due to privacy restrictions and the data protection law in Germany.
